# Sugar Content in Processed Foods in Spain and a Comparison of Mandatory Nutrition Labelling and Laboratory Values

**DOI:** 10.3390/nu12041078

**Published:** 2020-04-13

**Authors:** María José Yusta-Boyo, Laura M. Bermejo, Marta García-Solano, Ana M. López-Sobaler, Rosa M. Ortega, Marta García-Pérez, María Ángeles Dal-Re Saavedra, on behalf of the SUCOPROFS Study Researchers

**Affiliations:** 1 Spanish Food Safety and Nutrition Agency, Alcala, 56., 28014 Madrid, Spain; mgarcias@mscbs.es (M.G.-S.); mgarciape@mscbs.es (M.G.-P.); mdalre@mscbs.es (M.Á.D.-R.S.); 2 Nutrition and Food Science Department, Faculty of Pharmacy, Complutense University of Madrid, 28040 Madrid, Spain; mlbermej@ucm.es (L.M.B.); asobaler@ucm.es (A.M.L.-S.); rortega@ucm.es (R.M.O.); 3 VALORNUT Research Group, Faculty of Pharmacy, Complutense University of Madrid, 28040 Madrid, Spain

**Keywords:** nutrition labeling, food labeling, food processing, nutrition policy, Spain, food analysis, dietary sugars, reformulation

## Abstract

To reduce the sugar content of processed foods through reformulation, the first step is to determine the content of the largest sources of sugars in each country’s diet. The aim of this work was to describe the sugar content in the most commonly consumed processed foods in Spain and to compare that sugar’s labelling and laboratory analysis values (LVs and AVs, respectively) to confirm its adequacy. A sample of the 1173 most commonly consumed processed foods in Spain (28 groups; 77 subcategories) was collected. For each product, the total sugar content was compared according to its AV and LV. The median (25th –75th percentiles, interquartile range) sugar content by group was calculated for the total sample, and the groups were classified as “high sugar content” when this value was above 22.5 g/100g of product. The adequacy of the LV, according to the European Union (EU) tolerance requirements, was then evaluated, and each subcategory median was compared with the AV to determine its appropriateness via a median test for independent samples (*p* < 0.05). In total, 10 out of 28 groups presented high sugar content. Moreover, 98.4% of the products met the EU tolerance ranges. Finally, only one subcategory (“cured ham”) presented significant differences between the AV and LV median values (0.4 g vs. 0.1 g sugar/100g, *p* < 0.05). The groups of food products whose sugar content reduction could have the greatest impact on public health were identified. In addition, our study showed the high adequacy of LV with the EU labeling tolerance requirements, as well as the LV’s appropriateness as a tool to implement actions aimed at reducing sugar consumption.

## 1. Introduction

The prevalence of overweight, obesity, and related non-communicable diseases (cardiovascular diseases, diabetes, and cancer) remains high in all European countries, including Spain [1].

The impact of dietary risk factors on the mortality and morbidity associated with non-communicable diseases highlights the importance of implementing measures to improve the quality of citizens’ diets within national health policies [2].

Diets must meet energy needs and provide a variety of foods of a high nutritional quality that are safe to consume. Moreover, these diets should be sustainable, affordable, accessible, and culturally acceptable [3].

The European Union (EU) has long promoted initiatives to tackle obesity and to improve nutrition in European countries. One of the main initiatives of the European Commission was the adoption of the White Paper of 30 May 2007 entitled ‘A Strategy for Europe on Nutrition, Overweight and Obesity related health issues’ [4], focusing on actions that can be taken at the local, regional, national, and European levels. One of the initiatives included in this document is for the food industry (including retailers) to reformulate its products, particularly by reducing the content of salt, sugar, and fats.

In this regard, the High Level Group on Nutrition and Physical Activity (HLGNPA), composed of representatives of EU Member States and the European Commission, launched two EU Frameworks for the reformulation of food products: the EU Framework for National Initiatives on salt reductions [5] and the EU Framework for National Initiatives on Selected Nutrients [6] with two annexes: Annex I on saturated fats [7] and Annex II on added sugars [8]. These EU frameworks and annexes establish benchmarks and timelines for nutrient content reduction, focusing their action on certain food categories while taking into account the priorities, health needs, baseline nutrient contents, traditions, and pattern of consumption of each member state.

For more than a decade, the Ministry of Health in Spain, through the NAOS Strategy (Strategy for Nutrition, Physical Activity and the Prevention of Obesity) of the Spanish Agency for Food Safety and Nutrition (Spanish acronym AESAN, formerly Spanish Agency for Consumption, Food Safety and Nutrition, AECOSAN) has promoted reformulation initiatives for food and beverages, following the recommendations outlined in the HLGNPA Frameworks. To establish these initiatives, different studies have been carried out to determine the nutrient consumption and main food sources of the population and to ascertain the nutrient content (mainly fats and salt) of processed products [9,10,11,12,13]. The results of these studies have facilitated measures to reduce fats and salt in the main processed foods in Spain. Among these initiatives are the agreements between AESAN and food sector associations to achieve nutrient reduction targets, which were committed to all companies that belong to the sector association [14]. A successful example of public–private collaboration to achieve the reduction of salt content is the agreement (legal document) between AECOSAN, the Spanish Confederation of Bakers (CEOPAN by its Spanish acronym), and the Spanish Association of Manufacturers of Frozen Dough (ASEMAC by its Spanish acronym) signed in 2004, in which a salt reduction from 22 g NaCl/Kg in bread flour in 2004 to a maximum of 18 g NaCl/kg by 2008 was agreed upon. The average NaCl content measured in 2008 was 16.3 g of NaCl/Kg in bread-making flour [14]. In addition, a new study conducted in 2014 concluded that the salt content in bread in Spain has remained stable since 2008 [15].

However, no study has yet been conducted to ascertain the sugar content in processed products in Spain in order to establish reference values for addressing public health food policies, such as the reformulation or improvement of processed food and beverage composition.

For this reason, at the end of 2016, AESAN conducted the present study to describe the sugar content in the food groups included in the Annex II of added sugar [8] and in the most commonly consumed processed foods in Spain, especially by children and adolescents [16]. In addition, the other secondary objectives were to compare the label value (LV) with the laboratory analysis value (AV) in order to assess the adequacy of the LV based on the EU labelling requirements, and to study the appropriateness of using the label values as reference data to monitor the reformulation of sugar and other nutrients or for strategies such as front-of-pack labelling or marketing restrictions.

## 2. Materials and Methods

### 2.1. Sample Selection

We selected 28 food and beverage groups of processed foods to perform the present study (Table 1), starting with the 11 groups recommended in the Annex II (on added sugar) in the HLGNPA [8]. Some of these 11 groups were divided due to the diversity of products included in each of them. For example, “Sugary dairy products and other similar products” was divided into six groups: flavoured milk drinks, drinking yoghurt, yoghurt, dairy-based desserts, cheeses, and soy drinks. Moreover, according to the results observed in the ENALIA study (National Dietary Survey on the Child and Adolescent Population), the groups “milk”, “juices”, “nectars”, and “meat products” were included because these groups represent an important source of energy for children and adolescents in Spain [16].

The 28 processed food groups selected were classified into 77 subcategories according to their composition and legal denomination. For some analyses, classification by subcategory was used; this type of classification is considered more appropriate than classification by group due to the variability within a group. The number of products to be analyzed in each food subcategory was established by consensus among researchers. Food products were selected from among those with the greatest presence in the national market at that time (including both brand names and retailer brands), according to data published in the report on the Alimarket study in 2015 [17]. This report provided the most important economic and financial variables by sector in Spain, including information about 9286 companies in the food sector.

### 2.2. Data Collection

Once the groups and subcategories to be studied were selected, the total number of processed product samples was 1173. The study was awarded through public tender to “AENOR Laboratorio Alimentacion” (AENOR Food laboratory), which carried out the plans to purchase the products. All samples were acquired in October 2016 and transported under adequate storage conditions to “AENOR Laboratorio Alimentacion” to proceed with the storage and subsequent determination of the total sugar AV of each processed product. In addition, the total sugar LV declared in the mandatory nutrition labelling (MNL) was recorded.

The Luff–Schoorl method was used to measure the total sugar AV. This method is the official method to control sugars intended for human consumption in Spain [18] according to the European legislation First Commission Directive of 26 July 1979, which outlines the community methods for testing certain sugars intended for human consumption (79/786/EEC) [19]. This method involves the elimination of all reducing materials other than sugars present in the sample by drying; subsequently, the sugar content is assessed based on the reducing action in a cupro–alkaline solution.

In order to collect the total sugar LV, we considered the proposed methodology for the monitoring and food reformulation initiatives of the Joint Action on Nutrition and Physical Activity (JANPA) of the European Commission [20]. In this methodology, the MNL was accepted as the data source to collect nutritional information.

### 2.3. Statistical Analysis

The data collected were recorded in a database designed ad hoc (for this study). This procedure was carried out in other countries as a tool for the reformulation and monitoring of processed food and beverage composition (Oqali database) [21]. Statistical analyses were performed using the SPSS software (SPSS, version 25.0; SPSS, Chicago, IL, USA).

For a descriptive study of the total sugar AV and LV, the median (25th–75th percentiles, interquartile range) were calculated for each food group, in the total sample (*n* = 1173), and in the two subsamples, according to the presence of nutrition claims about sugar content (light, low sugar content, no added or zero sugar, etc.) on the label (*n* = 64) vs. the absence of a sugar-based nutritional claim (*n* = 1109). Moreover, an adequacy study of the LV based on the EU labelling requirements, using the tolerance of the values declared on the labelling [22], was conducted. The final sample used to study adequacy of the LV included 1074 products (excluding products with nutritional claims and without a LV). A product met the tolerance range (“Meets”) when its LV < 10 g/100g of product and its deviation from the AV was ±2 g; when its LV was 10–40 g/100 g and its deviation from the AV was ±20%; or when its LV > 40 g/100 g and deviation from the AV was ±8 g. Any product outside of these tolerance ranges was classified as “Does not meet”.

Finally, an appropriateness study of LV as reference data for reformulation, monitoring, and other strategies was conducted. For the sample consisting of 1074 products, the LV and AV medians of each product subcategory were compared using the SPSS Median Test for 2 Independent Medians (*p* < 0.05).

## 3. Results

**Figure 1:**
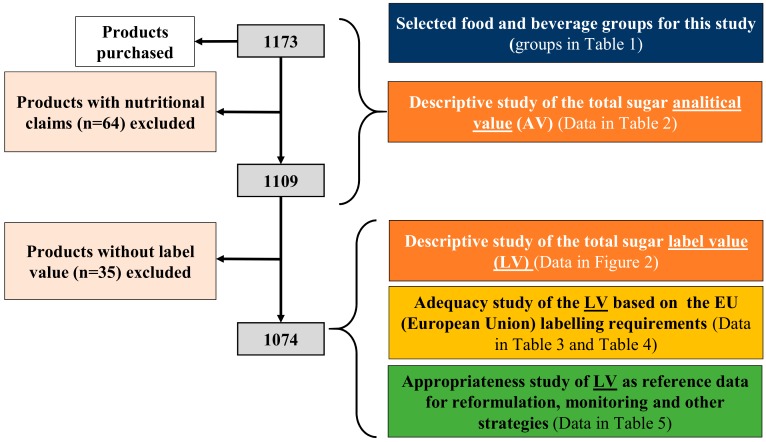
Flow diagram. Sample selection and the different analyses carried out.

A flowchart with the sample selection and the different analyses carried out in the present study is detailed in Figure 1.

### 3.1. Descriptive Study of the Total Sugar AV and LV

Table 2 provides the results of the AV statistical analysis median (25th–75th percentiles, interquartile range) of the total sample analyzed (*n* = 1173). In addition, the analysis of the subsamples according to the presence or absence of a nutrition claim about sugar content showed that the total sugar AV was higher in products without a nutritional claim (*n* = 1109) than in those with such a claim (*n* = 64).

**Figure 2:**
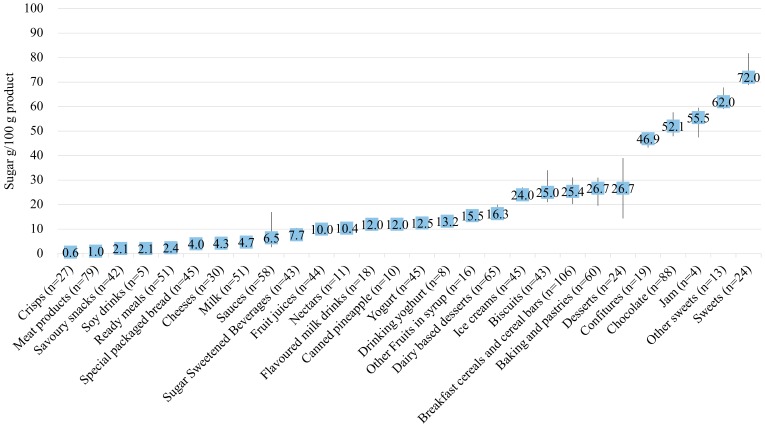
Median and 25th and 75th percentiles of label values (LV) for total sugar for the most consumed groups of products in Spain.

Figure 2 presents the median content of the total sugar LV for each group of products studied in the total sample. The 25th-75th percentiles and interquartile ranges (IQRs) are also shown.

Moreover, according to data from the AV of products without nutritional claims (*n* = 1109), the food groups were classified as high total sugar content groups (*n* = 10) and no high total sugar content groups (*n* = 18), with the value 22.5 g/100 g as the cut-off point according to the criterion used for the front of the pack in the UK (25% of the recommended intake in Annex XIII of the EU regulation 1169/2011) [23] and in Chile’s Law on Food Labelling and Advertising [24]. There were a total of 10 groups with high total sugar content (in descending order): sweets, other sweets, jam, chocolate, confitures, baking and pastries, desserts, breakfast cereals and cereal bars, biscuits, and ice creams. For the dispersion within each group, the estimated interquartile range was greater than 15 g/100 g in three groups (desserts, sauces, and sweets), between 15 and 10 g/100 g in six groups (biscuits, other sweets, jam, breakfast cereals and cereal bars, baking and pastries, and chocolate), and lower than 10 g/100g in 19 groups.

### 3.2. Adequacy Study of the LV Based on the EU Labelling Requirements for the Tolerance of the Values Declared on the Label

An adequacy study of the LV compared to the EU labelling requirements was conducted considering the tolerance for the values declared on the label [22]. Tolerance refers to the acceptable difference between the nutritional values declared on the label and those established over the course of official controls in relation to the “nutritional information” or “nutritional labeling” described in Regulation (EU) No. 1169/2011 on the provision of food information provided to consumers [25].

For this study, 64 products with nutritional claims related to sugar content were excluded (Table 2) since their sugar content was reduced to comply with the requirements of Regulation (EC) No. 1924/2006 on the nutritional and health claims made for foods [26], the tolerance requirements for these products are different and are beyond the scope of this study, which focuses instead on products that can be reformulated. In addition, 35 products without LV data (0.09% of the total samples analyzed) were also excluded (11 meat products, five sweets, four ready meals, four from other sweets, three crisps, three savoury snacks, two chocolates, one sauce, one dessert, and one fruit in syrup). Notably, the obligation to show the mandatory nutrition labelling (MNL) (according to Regulation (EU) No. 1169/2011 on the food information provided to the consumer) has been in force since 13 December 2016 [25], but the sampling for this study took place earlier (October 2016). Therefore, the final sample included 1074 products. Therefore, the final sample analyzed was 1074 products.

Of the aforementioned 1074 products, 1057 (98.4%) met the tolerance ranges, while 17 products did not, among which only five (0.45% of the total sample) declared a total sugar LV in the NML that was lower than an AV: one in the “baking and pastries” group (*n* = 60; 1.7% of the group’s products), one in the “breakfast cereals and cereal bars” group (n = 106; 0.9%), one in the “special packaged bread” group (*n* = 45; 2.2%), one in the “desserts” group (n = 24; 4.2%), and one in the “dairy-based desserts” group (*n* = 65; 1.5%), while the remaining 12 (1.1% of the total sample) declared a value in their NML greater than the AV (Table 3).

Table 4 shows the 17 products that did not meet the tolerance ranges.

### 3.3. Appropriateness Study of Using the LV as Reference Data for Reformulation, Monitoring, and other Strategies

Table 5 shows the medians and the 25th and 75th percentiles of the LV and AV data obtained for each group and subcategory of products. Of the 28 groups studied, only the “meat products” group presented significant differences between both medians, with the LV being greater than the AV (LV: 1.0 (0.0–5.0) g vs. AV: 0.7 (0.1–4.2) g, *p* < 0.05). In addition, in the study by subcategories, within the group of meat products, “cured ham” was the only subcategory that presented significant differences (LV: 0.4 g vs. AV: 0.1 g, *p* < 0.05).

## 4. Discussion

To study the content of total sugar, we presented the AVs of 28 groups of processed food products that are most frequently consumed by the Spanish population. The groups whose reformulation could have the greatest impact on public health were identified by their high sugar content and dispersion, which indicates that there is room for the reduction of their sugar content and that reformulation is, therefore, possible.

The most commonly consumed groups, especially by children and adolescents, should also be distinguished and prioritized (compared to those consumed more rarely) based on their energy contribution to the diet (cereals and meats and derivatives, among others), added sugar contribution to the diet (sugar sweetened beverages, chocolate, and nectars), or both (dairy products, baking and pastries, and breakfast cereals, among others) [16,27,28]. In Europe, high sugar consumption, especially in children and adolescents [29], has become a major public health concern, which is highlighted by scientific reports and studies associating high sugar consumption with an increased risk of dental caries [30], overweight [31], cardiometabolic risk factors [32], and adult cardiovascular mortality [33].

This study, promoted by the Observatory of Nutrition and Study of Obesity of AESAN in the framework of the NAOS Strategy, answers the call to action of the EU from the Council Conclusions of June 2016 for the improvement of food to develop a national reformulation initiative (the “Collaboration Plan for the improvement of the composition of food and beverages and other measures 2020” [34]), which is in line with the EU Plan of Action against Childhood Obesity 2014–2020 [35] and the WHO European Action Plan for Food and Nutrition 2015–2020 [36].

One of the limitations of this study is its small sample size, which was mainly due to our limited budget. For some subcategories, this reduced sample size was due to other specific reasons. For “pineapple in syrup”, the market is dominated by a single brand, and for “beverages with fruits” (included in the group “sugar sweetened beverages”), we decided to create a new subcategory, resulting in only three products; sugar content in fruit is very different from that in the rest of the group, so the groups could not all be considered together. For this reason (sample size) and to prevent the influence of the most distal values, the median was used as a measure of the central tendency. Moreover, to produce a snapshot of sugar availability in our food environment and to estimate the potential impact of reformulation, market shares should have been considered. In our more feasible approach, the products in each subcategory were selected according to the results of a food market study that identified the most commonly consumed food products.

Our results provide novel information on the presence of total sugars in our food environment. This study quantifies the total sugar content in the main groups of processed products on the Spanish market, assesses the adequacy of label values based on the tolerance ranges for sugar according to EU labelling requirements, and assesses the appropriateness of using the LV as reference data. These are relevant aspects for designing and implementing actions aimed at reducing sugar consumption, which will help tackle obesity and its consequences for health.

Reformulation policies aimed at reducing the content of certain nutrients are some of the measures recommended by international institutions and organizations (European Union, WHO, OECD) to improve the quality of diets, reduce the consumption of foods high in salt, fats, and sugars, and prevent obesity and its related non-communicable diseases [37].

The Annex II on added sugars [8] proposes that the Member States should set a general benchmark for a minimum of 10% added sugar reduction in food products against their baseline levels or to move towards a ‘best in class’ level of sugar content.

In Spain, in order to establish the sugar reduction objectives of the “Collaboration plan for the improvement of the composition of food and beverages and other measures 2020” [34], AESAN opted in 2017 to deploy the first strategy mentioned in Annex II, which entails the reduction of a percentage of sugar from the basal median content, which was considered a viable and realistic method. Thus, all companies in the sector related to each food subcategory made a commitment to follow this plan. The evaluation of this plan, after its completion in 2020, will allow us to determine the degree of compliance with the objectives and to draw pertinent conclusions about the contribution of this initiative and its possible “drag effect” on other subsequent initiatives.

This work shows that most of the analyzed processed products (98.4%) meet the European Union tolerances for nutrient values declared on the labels. A very low percentage of products in our study did not meet the tolerance values because they had a lower LV than AV (0.45%). A study conducted with a sample of products that contribute the most to sodium intake in the United States [38] concluded that the majority of the labeling and analytical values agree with each other; thus, label under-declaration is limited. However, the authors observed that the differences in total sugars were greater and more systematic and that 19% products did not meet tolerance requirements because their labelled total sugars were lower than their analytical data. In our study, each of the five products that did not meet tolerance levels (due to a lower LV than AV) belonged to a different group (baking and pastries, breakfast cereals, special packaged bread, desserts, and dairy-based desserts), which also included a considerable number of other products that did meet the tolerance requirements.

To our best knowledge, this is the first time that the LVs and AVs of the studied processed products groups were compared in Spain, showing that the total sugar values are similar under both methods for most of the products and subcategories, according to the tolerance requirements for the nutritional information established by the European Union. Of the 28 food and beverage groups and the 77 subcategories analyzed, only the “meat products” group and, specifically, the “cured ham” subcategory, showed significant differences between their median AV and LV data. Taking into account the low sugar content of cured ham and the specific characteristics of its manufacturing and maturation process, such as the infiltration variability of additives depending on the part of the product (fat, bone, etc.), these differences are not considered relevant.

In light of these findings, we conclude first that a reduction in sugar content is feasible in a wide range of products. We recommend setting benchmarks at the subcategory level because this level includes similar products to which the same quality standards apply and allows one to compare the nutrient content of each product within a subcategory to its median, thus facilitating the identification of products with the greatest potential for reformulation.

Moreover, our results show a remarkably high compliance with the tolerance requirements and the appropriateness of the declared total sugar content in the MNL for most sold packaged processed products in Spain.

Thus, the MNL provides an accessible and efficient tool for various aims: to inform consumers truthfully, to conduct studies on the sugar content or other nutrients in labeled products, to establish and monitor reformulation initiatives, to implement front-of-pack initiatives, to apply nutritional profiles for different objectives, and for food advertising policies, among others. Regulation (EU) No. 1169/2011 [25] establishes (starting from 13 December 2016) the mandatory obligation to provide a nutritional declaration on the labels of most processed food products. This declaration must include the energy values and six nutrients, one of them being the sugar content.

Briefly, the groups identified to boost reformulation policies based on their sugar content, the differences in sugar content between similar products, and their contributions to energy, added sugars, or both to the diet are the following: sweets, other sweets, jam, chocolate, confitures, desserts, baking and pastries, breakfast cereals and cereals bars, biscuits, ice cream, sauces, meat products, sugar sweetened beverages, nectars, and dairy products [16,27,28].

The results obtained from this research may help make the nutritional composition of food products more visible, to better explore the feasibility of improving nutrition and evaluate related actions.

## 5. Conclusions

The results of the present study will help identify the groups of food products whose sugar content reduction could have the greatest impact on public health. In addition, we showed the adequacy of labelling values with the EU labelling tolerance requirements; labelling values are, therefore, an adequate tool to implement and evaluate actions aimed at reducing sugar consumption.

## Figures and Tables

**Table 1:** Selected food and beverage groups for this study.

Food Groups to Focus Action on Added Sugar Reduction, According to Annex II of the High Level Group on Nutrition and Physical Activity (HLGNPA)	Selected Groups for this Study (HLGNPA Groups + other Highly Consumed Groups by Spanish Children)
Sugar sweetened Beverages	Sugar sweetened beverages
Sugar sweetened dairy and dairy imitates	Flavoured milk drinks
	Drinking yoghurt
	Yoghurt
	Dairy-based desserts
	Cheese
	Soy drinks
Breakfast cereals	Breakfast cereals and cereal bars
Bread and bread products	Special packaged bread
Bakery products (e.g., cakes and cookies)	Baking and pastries
	Biscuits
Confectionaries	Sweets
	Chocolates
	Other sweets (chewing gum, marshmallow, etc.)
Ready meals (including ready to prepare products like dry soups, dried mashed potatoes, rice mixture)	Ready meals
Savoury snacks	Savoury snacks
	Crisps
Sauces (including ketchup)	Sauces
Sugar sweetened desserts, ice cream and topping	Desserts
	Ice creams
	Jam
	Confitures
Canned fruits and vegetables	Canned pineapple
	Other Fruits in syrup
	Milk
	Fruit juices
	Nectars
	Meat products

**Table 2:** Analytical value (AV) of the total sugar content (g/100g of product) in the different groups of products, in the total sample, and in the two subsamples according to the presence or absence of nutritional claims related to sugar content.

	All Products (*n* = 1173)			Products with Nutritional Claims (*n* = 64)			Products without Nutritional Claims (*n* = 1109)		
Groups	*n*	Median	(P25–P75) IQR	N	Median	(P25–P75) IQR ^2^	n	Median	(P2–P75) IQR
Baking and pastries	61	27.3	(19.8–31.5) 11.7	1	--	4.6	60	27.4	(19.9–31.7) 11.9
Biscuits	45	24.9	(20.9–34.0) 13.1	2	--	(0.3, 0.6)	43	25.3	(21.0–34.1) 13.1
Breakfast cereals and cereal bars	107	25.8	(19.3–31.4) 12.1	1	--	0.1	106	25.9	(19.3–31.4) 12.1
Canned pineapple	10	11.7	(11.4–12.2) 0.8	--	--	--	10	11.7	(11.4–12.2) 0.8
Cheeses	30	4.4	(2.8–4.9) 2.1	--	--	--	30	4.4	(2.8–4.9) 2.1
Chocolate	92	52.0	(47.3–58.0) 10.7	2	--	(0.6, 13.5)	90	52.6	(47.8–58.0) 10.2
Confitures	30	42.5	(26.3–46.7) 20.4	11	5.5	(2.2–35.0) 32.8	19	46.5	(42.6–47.0) 4.4
Crisps	30	0.7	(0.4–0.8) 0.4	--	--	--	30	0.7	(0.4–0.8) 0.4
Dairy-based desserts	65	15.9	(14.1–19.3) 5.2	--	--	--	65	15.9	(14.1–19.3) 5.2
Desserts ^1^	29	23.2	(12.7–33.8) 21.1	4	9.8	(6.7–23.1) 16.4	25	26.7	(15.6–39.1) 23.5
Drinking yoghurt	10	13.1	(11.6–13.2) 1.6	2	--	(4.0, 4.7)	8	13.2	(12.7–13.4) 0.7
Flavoured milk drinks	20	11.3	(10.4–12.0) 1.6	2	--	(4.6, 5.8)	18	11.6	(10.9–12.0) 1.1
Fruit juices	44	10.0	(9.0–11.0) 2.1	--	--	--	44	10.0	(9.0–11.0) 2.1
Ice creams	45	24.2	(21.0–27.1) 6.1	--	--	--	45	24.2	(21.0–27.1) 6.1
Jam	5	51.2	(43.2–58.8) 15.6	1	--	2.9	4	55.0	(47.2–59.5) 12.3
Meat products	90	0.7	(0.1–1.2) 1.1	--	--	--	90	0.7	(0.1–1.2) 1.1
Milk	51	4.7	(4.6–4.8) 0.2	--	--	--	51	4.7	(4.6–4.8) 0.2
Nectars	18	9.5	(4.3–10.7) 6.4	7	4.2	(4.1–6.0) 1.9	11	10.4	(9.6–11.1) 1.5
Other Fruits in syrup	20	14.3	(13.7–16.4) 2.7	3	5.3	(5.2–5.8) 0.6	17	15.6	(14.0–16.7) 2.7
Other sweets	27	49.4	(0.1–66.9) 66.8	10	0.1	(0.1–0.1) 0.0	17	61.6	(54.8–67.1) 12.3
Ready meals	55	2.1	(1.0–3.2) 2.2	--	--	--	55	2.1	(1.0–3.2) 2.2
Sauces	63	6.4	(2.4–17.0) 14.6	4	3.7	(2.5–7.6) 5.1	59	6.4	(2.4–18.9) 16.5
Savoury snacks	45	1.2	(0.4–2.8) 2.4	--	--	--	45	1.2	(0.4–2.8) 2.4
Soy drinks	5	2.1	(1.0–2.7) 1.7	--	--	--	5	2.1	(1.0–2.7) 1.7
Special packaged bread	45	4.4	(3.9–5.1) 1.2	--	--	--	45	4.4	(3.9–5.1) 1.2
Sugar Sweetened Beverages	47	7.2	(4.7–10.1) 5.4	4	4.6	(4.0–5.8) 1.9	43	7.6	(6.0–10.2) 4.2
Sweets	34	70.2	(62.8–76.1) 13.3	5	0.1	(0.1–0.1) 0.0	29	71.4	(66.3–81.7) 15.4
Yoghurt	50	12.3	(5.8–13.5) 7.7	5	4.9	(4.9–5.2) 0.3	45	12.6	(10.5–13.6) 3.1
Total	1173	11.6	(3.7–26.9) 23.2	64	4.2	(0.2–5.8) 5.6	1109	12.2	(4.0–28.1) 24.1

Including non-dairy desserts (jello, “tocino de cielo” (pudding made with egg yolks and syrup), and chocolate cake) and powders for dessert preparation (flan powder, cake powder, and chocolate cake powder). ^2^ For groups with 1 or 2 items, the median was not calculated, and the data are presented in the IQR column separated by a comma; IQR = Interquartile range.

**Table 3:** Products that meet or do not meet the EU tolerance ranges.

		Meets *n* (%)	Does not Meet (*n* (%))	
	*n*		Label Value (LV) >Analytical Vvalue (AV)	Label value (LV) <Analytical value (AV)
Baking and pastries	60	59 (98.3)	0 (0)	1 (1.7)
Biscuits	43	43 (100)	0 (0)	0 (0)
Breakfast cereals and cereal bars	106	102 (96.2)	3 (2.8)	1 (0.9)
Canned pineapple	10	10 (100)	0 (0)	0 (0)
Cheese	30	29 (96.7)	1 (3.3)	0 (0)
Chocolate	88	88 (100)	0 (0)	0 (0)
Confitures	19	19 (100)	0 (0)	0 (0)
Crisps	27	27 (100)	0 (0)	0 (0)
Dairy-based desserts	65	64 (98.5)	0 (0)	1 (1.5)
Desserts	24	23 (95.8)	0 (0)	1 (4.2)
Drinking yoghurt	8	8 (100)	0 (0)	0 (0)
Flavoured milk drinks	18	18 (100)	0 (0)	0 (0)
Fruit juices	44	44 (100)	0 (0)	0 (0)
Ice creams	45	45 (100)	0 (0)	0 (0)
Jam	4	4 (100)	0 (0)	0 (0)
Meat products	79	78 (98.7)	1 (1.3)	0 (0)
Milk	51	51 (100)	0 (0)	0 (0)
Nectars	11	10 (90.9)	1 (9.1)	0 (0)
Other fruits in syrup	16	16 (100)	0 (0)	0 (0)
Other sweets	13	13 (100)	0 (0)	0 (0)
Ready meals	51	51 (100)	0 (0)	0 (0)
Sauces	58	57 (98.3)	1 (1.7)	0 (0)
Savoury snacks	42	39 (92.9)	3 (7.1)	0 (0)
Soy drinks	5	5 (100)	0 (0)	0 (0)
Special packaged bread	45	44 (97.8)	0 (0)	1 (2.2)
Sugar Sweetened Beverages	43	41 (95.3)	2 (4.7)	0 (0)
Sweets	24	24 (100)	0 (0)	0 (0)
Yoghurt	45	45 (100)	0 (0)	0 (0)
Total	1074	1057 (98.4)	12 (1.1)	5 (0.5)

**Table 4:** Individual label values (LVs) and analytical values (AVs) of the products that did not meet EU tolerance ranges.

Groups	Product	LV (g/100g)	AV (g/100g)	LV-AV (g/100 g)
Baking and pastries	Children’s industrial bakery	20	38.6	−18.6
Breakfast cereals and cereal bars	Breakfast cereals with honey	49	35	14
	Cereal bars	36	21	15
	Integral breakfast cereals with fruit	23	30.5	−7.5
	Muesli	20.2	12.9	7.3
Cheeses	Spread and melted cheeses	7	4.7	2.3
Dairy-based desserts	Custard	4.5	15.2	−10.7
Desserts	Chocolate cake	15.2	21.9	−6.7
Meat products	Chopped	3.5	0.8	2.7
Nectars	Nectar	14.4	10	4.4
Sauces	Mustard	5.9	3.5	2.4
Savoury snacks	Microwave popcorn 1	3.8	0.4	3.4
	Microwave popcorn 2	3.8	0.8	3
	Microwave popcorn 3	3.4	0.4	3
Special packaged bread	Integral tin loaf bread	3	6.2	−3.2
Sugar Sweetened Beverages	Sugar sweetened beverage 1	6.3	4.1	2.2
	Sugar sweetened beverage 2	5.2	3.1	2.1

**Table 5:** Comparison of the label value (LV) and analytical value (AV) medians of the total sugar for each group and subcategory of products (appropriateness study).

Groups	Subcategories	*n*	Label Value (LV) (g/100 g)	Analytical Value (AV) (g/100 g)
			Median (P25–P75)	Median (P25–P75)
Baking and pastries	Industrial croissants and similar	11	12.0 (12.0–13.0)	12.4 (12.1–13.0)
	Industrial pastries for children	21	32.0 (24.6–39.0)	32.0 (26.5–38.0)
	Muffins	15	29.0 (26.4–30.0)	29.1 (26.9–31.3)
	Other (donuts, etc.)	13	24.4 (19.0–30.0)	24.4 (19.8–29.9)
	Total	60	26.7 (19.5–31.0)	27.4 (19.9–31.7)
Biscuits	Filled biscuits	18	37.0 (32.0–41.0)	37.3 (33.1–40.7)
	Sweet biscuit	21	22.0 (21.0–24.0)	23.6 (20.9–24.6)
	Other unfilled biscuits (digestives, cookies, etc.)	4	18.0 (17.2–18.0)	18.0 (17.2–18.2)
	Total	43	25.0 (21.0–34.0)	25.3 (21.0–34.1)
Breakfast cereals and cereal bars	Breakfast Cereals with honey	16	30.5 (26.7–36.0)	31.0 (27.5–35.1)
	Cereal bars	16	30.7 (28.0–36.0)	30.4 (27.5–34.5)
	Chocolate filled Breakfast Cereals	6	33.3 (29.0–36.0)	34.3 (29.4–36.8)
	Chocolate flavoured Breakfast Cereals	14	28.9 (28.0–34.0)	29.7 (28.7–34.0)
	Cornflakes cereals	14	7.0 (5.0–8.0)	7.1 (4.9–8.0)
	Muesli	16	22.2 (20.2–25.7)	22.4 (18.9–24.3)
	Sugared breakfast cereals	12	25.0 (24.1–30.5)	24.4 (23.4–30.0)
	Other breakfast cereals	12	19.0 (2.8–23.5)	18.8 (2.8–23.5)
	Total	106	25.4 (20.2–31.0)	25.9 (19.3–31.4)
Canned pineapple	Canned pineapple	10	12.0 (11.6–12.0)	11.7 (11.4–12.2)
	Total			
Cheeses	Spread and melted cheeses	30	4.3 (3.0–5.2)	4.4 (2.8–4.9)
	Total			
Chocolates	Chocolate bars	15	49.5 (44.0–51.1)	48.9 (46.3–51.7)
	Chocolate eggs and similar	5	57.7 (57.6–58.0)	58.0 (56.9–58.2)
	Chocolate large bars (dark, with milk, white)	31	53.8 (46.0–55.9)	54.0 (46.3–56.1)
	Chocolate like bean (carob) and similar	5	53.8 (43.7–64.1)	53.5 (44.4–62.4)
	Chocolate spreads	5	58.0 (57.0–59.0)	58.0 (56.8–58.1)
	Chocolates	20	49.6 (47.1–51.9)	50.3 (46.7–51.8)
	Cocoa powder	7	70.0 (67.0–75.7)	69.9 (68.1–75.4)
	Total	88	52.1 (47.9–57.7)	52.0 (47.7–58.0)
Confitures	Confitures	19	46.9 (43.2–47.0)	46.5 (42.6–47.0)
	Total			
Crisps	Crisps	27	0.6 (0.5–0.9)	0.7 (0.4–0.8)
	Total			
Dairy-based desserts	Custard	15	16.0 (15.0–16.8)	15.9 (15.0–16.6)
	Flan	15	20.8 (16.0–24.3)	19.7 (15.9–23.3)
	Flavoured fromage frais	13	13.4 (13.0–14.0)	13.1 (12.3–13.8)
	Others (chocolate cups, mousse, etc.)	22	17.4 (16.0–20.0)	17.4 (15.0–19.9)
	Total	65	16.3 (14.9–20.0)	15.9 (14.1–19.3)
Desserts	Non-dairy desserts	16	19.6 (14.4–35.0)	21.0 (14.3–36.0)
	Powder for dessert preparation ^1^	8	29.0 (13.3–70.4)	28.9 (13.6–69.9)
	Total	24	26.7 (14.4–39.0)	26.9 (14.3–39.6)
Drinking yoghurt	Drinking yoghurt	8	13.2 (12.7–13.8)	13.2 (12.7–13.4)
	Total			
Flavoured milk drinks	Flavoured milk drinks	18	12.0 (11.0–12.0)	11.6 (10.9–12.0)
	Total			
Fruit juices	Fruit juices	44	10.0 (9.2–11.0)	10.0 (9.0–11.0)
	Total			
Ice creams	Ice cream to share (bars, frozen cakes, etc.)	25	24.0 (22.6–26.0)	23.8 (21.0–26.2)
	Individual ice cream	20	25.4 (21.5–29.0)	25.0 (21.1–28.4)
	Total	45	24.0 (22.0–27.0)	24.2 (21.0–27.1)
Jam	Jam	4	55.5 (47.4–59.5)	55.0 (47.2–59.5)
	Total			
Meat products	Chopped	9	1.0 (0.5–2.5)	0.8 (0.1–0.9)
	Cooked ham	10	1.1 (1.0–1.3)	0.9 (0.7–1.4)
	Cured ham	15	0.4 (0.1–0.5)	0.1 (0.1–0.4)*
	Cured sausage (chorizo)	9	0.5 (0.3–1.0)	0.4 (0.1–1.0)
	Cured sausage (salchichon)	9	3.0 (1.6–3.5)	3.0 (1.7–3.8)
	Sausages	18	1.0 (0.5–1.0)	0.7 (0.4–1.2)
	Turkey	9	1.0 (0.4–2.0)	0.5 (0.1–1.5)
	Total	79	1.0 (0.5–1.6)	0.7 (0.1–1.3)*
Milk	Whole milk	15	4.6 (4.6–4.7)	4.7 (4.6–4.8)
	Semi-skimmed milk	15	4.7 (4.7–4.8)	4.7 (4.6–4.7)
	Skimmed milk	15	4.8 (4.7–4.8)	4.7 (4.7–4.8)
	Lactose free milk	6	4.8 (4.7–4.8)	4.7 (4.6–4.8)
	Total	51	4.7 (4.7–4.8)	4.7 (4.6–4.8)
Nectar	Nectar	11	10.4 (10.0–11.6)	10.4 (9.6–11.1)
	Total			
Other fruits in syrup	Peach	8	16.2 (14.0–17.0)	15.9 (13.9–17.1)
	Pineapple	3	15.0 (14.0–16.4)	14.2 (14.1–16.7)
	Other fruits	5	14.0 (14.0–16.0)	14.3 (13.8–15.6)
	Total	16	15.5 (14.0–16.4)	15.0 (13.9–16.4)
Other sweets	Other sweets ^2^	13	62.0 (59.0–67.8)	62.0 (59.3–67.5)
	Total			
Ready meals	Lasagna /cannelloni	11	2.7 (1.2–3.4)	2.7 (1.0–3.4)
	Pizza	20	2.4 (1.7–3.5)	2.7 (1.7–3.4)
	Others	20	2.0 (1.0–3.0)	1.9 (0.8–2.8)
	Total	51	2.4 (1.2–3.1)	2.3 (1.3–3.3)
Sauces	Ketchup	14	21.4 (19.3–22.8)	20.8 (19.1–22.8)
	Mayonnaise	14	1.6 (1.4–3.0)	1.4 (1.0–1.9)
	Tomato sauce	13	7.4 (6.7–8.1)	7.2 (7.1–7.8)
	Other sauces	17	3.0 (2.6–5.9)	3.2 (2.6–4.9)
	Total	58	6.5 (2.6–17.0)	6.4 (2.4–17.0)
Savoury snacks	Corn snacks	15	2.2 (1.0–4.1)	2.1 (0.8–4.2)
	Microwave popcorn	8	1.1 (0.4–3.6)	0.4 (0.4–0.9)
	Other savoury snacks ^3^	19	1.8 (0.7–5.1)	1.9 (0.1–4.9)
	Total	42	2.1 (0.8–3.8)	1.4 (0.4–3.7)
Soy drinks	Soy drinks	5	2.1 (0.7–2.8)	2.1 (1.0–2.7)
	Total			
Special packaged bread	White tin loaf bread	14	3.2 (2.9–4.0)	4.0 (3.5–4.5)
	Integral tin loaf bread	16	3.0 (2.7–4.2)	4.2 (3.4–4.8)
	Toasted bread	15	5.1 (4.3–5.6)	5.5 (4.4–5.9)
	Total	45	4.0 (3.0–5.0)	4.4 (3.9–5.1)
Sugar sweetened beverages	Beverages with fruits	3	11.0 (4.6–11.9)	10.8 (4.6–11.9)
	Sugar Sweetened beverages	40	7.5 (6.3–10.2)	7.5 (6.1–10.1)
	Total	43	7.7 (6.3–10.5)	7.6 (6.0–10.2)
Sweets	Sweets	24	72.0 (68.8–81.8)	71.9 (67.2–81.9)
	Total			
Yoghurt	Plain yoghurt	9	4.0 (4.0–4.3)	4.0 (3.9–4.8)
	Flavoured yoghurt	18	12.8 (11.0–14.0)	12.8 (11.4–13.5)
	Fruit Yoghurt	12	14.1 (12.8–15.0)	14.3 (12.8–15.1)
	Sugar sweetened yoghurt	6	12.5 (12.3–13.3)	12.4 (12.2–13.4)
	Total	45	12.5 (10.1–14.0)	12.6 (10.5–13.6)

* Significant differences between the labelling value (LV) and laboratory analysis value (AV) (median test for unpaired samples); ^1^ Powder for dessert preparation (flan powder, cake powder, chocolate cake powder, etc.); ^2^ Sweet gels, liquorice, marshmallow, chewing gum; ^3^ Wheat rinds, pork rinds, potato sticks, crackers.
